# Predisposing factors for allogeneic blood transfusion in patients with ankylosing spondylitis undergoing primary unilateral total hip arthroplasty: a retrospective study

**DOI:** 10.1186/s13018-022-03464-z

**Published:** 2023-01-04

**Authors:** Tao Bian, Liang Zhang, Siliang Man, Hongchao Li, Yong Dou, Yixin Zhou

**Affiliations:** 1 Department of Orthopedic Surgery, Beijing Jishuitan Hospital, Fourth Clinical College of Peking University, No. 31 Xinjiekou East Street, Xicheng District, Beijing, 100035 China; 2 Department of Rheumatology and Immunology, Beijing Jishuitan Hospital, Fourth Clinical College of Peking University, No. 31 Xinjiekou East Street, Xicheng District, Beijing, 100035 China

**Keywords:** Allogeneic blood transfusion, Ankylosing spondylitis, Hemoglobin, Hip arthroplasty, Tranexamic acid

## Abstract

**Background:**

The transfusion rate is relatively high in patients with ankylosing spondylitis (AS) undergoing total hip arthroplasty (THA). However, relevant studies focusing on the predisposing factors for transfusion with a large sample size are lacking. This study aimed to investigate the incidence of and risk factors for allogeneic blood transfusion in patients with AS undergoing primary unilateral THA.

**Methods:**

This retrospective study included 331 patients with AS who underwent primary unilateral THA between 2011 and 2021. Relevant parameters were collected through a chart review. Multivariate logistic regression analysis was conducted to identify possible factors associated with perioperative allogeneic blood transfusion.

**Results:**

A total of 113 (34.1%) patients received perioperative allogeneic blood transfusions. Factors related to receiving an allogeneic blood transfusion included prolonged operative duration (odds ratio [OR] per 10 min = 1.139, *P* = 0.047), increased estimated intraoperative blood loss (OR per 100 mL = 1.348, *P* < 0.001), and increased postoperative drainage volume (OR per 100 mL = 1.235, *P* = 0.024). A higher body mass index (BMI) (OR = 0.914, *P* = 0.012), perioperative tranexamic acid (TXA) use (OR = 0.166, *P* < 0.001), and a higher preoperative hemoglobin level (OR per 1 g/dL = 0.744, *P* = 0.004) decreased the risk of transfusion.

**Conclusions:**

In patients with AS undergoing THA, prolonged operative duration, increased estimated intraoperative blood loss, and increased postoperative drainage volume were found to be risk factors for transfusion, whereas a higher BMI, perioperative TXA use, and a higher preoperative hemoglobin level were protective factors. These results may aid in developing a better perioperative management strategy, ultimately reducing the need for transfusion.

## Background

Ankylosing spondylitis (AS) is a chronic inflammatory disease that commonly involves the hip joint [1]. Total hip arthroplasty (THA) for patients with AS and end-stage hip involvement is an effective method to alleviate pain and improve function, with good surgical and survival outcomes [2, 3]. Unfortunately, THA is associated with excessive blood loss, and 73.3–87.5% of patients with AS end up requiring perioperative transfusion [4–6].

Previous studies have identified risk factors for allogeneic blood transfusion during primary THA, such as female sex, low preoperative hemoglobin level, prolonged operative duration, increased intraoperative blood loss, and drainage use [7, 8]. Blood transfusion is associated with higher mortality and infection rates, longer length of hospitalization, and increased total cost of hospital admission [9–12]. However, previous studies focusing on patients with AS had a relatively small sample size, impeding the identification of independent risk factors for transfusion [4–6]. Consequently, the identification of relevant risk factors is crucial to preoperatively optimize modifiable factors and reduce the need for transfusion.

This study aimed to investigate the incidence of and identify predisposing factors for perioperative allogeneic blood transfusion in patients with AS undergoing primary unilateral THA.

## Methods

A total of 331 patients with AS who underwent THA between 2011 and 2021 were enrolled in this retrospective study. We included patients with a diagnosis of AS according to the modified New York criteria [13] who underwent primary unilateral THA using a posterolateral approach. Patients who underwent staged bilateral THA were only included if the operation interval was at least 3 months. We excluded patients who underwent simultaneous bilateral THA or revision surgery; those with a history of infection, trauma, or surgery in the operated hip; those with known coagulation diseases; and those who had undergone autologous blood pre-donation. This study was approved by the Institutional Review Board of our hospital (Ethics Approval Number: 202004-83). The requirement for informed consent from patients was waived owing to the retrospective nature of this study.

Demographic data included sex, age at THA, and body mass index (BMI). Clinical parameters included disease duration, Bath Ankylosing Spondylitis Disease Activity Index, Bath Ankylosing Spondylitis Functional Index [14], Harris hip score, American Society of Anesthesiologists score, and the presence or absence of bony ankylosis. Operation-related data included perioperative tranexamic acid (TXA) use, type of anesthesia, operative duration, estimated intraoperative blood loss, allogeneic transfusion volume, wound blood salvage transfusion volume, and drainage volume.

The preoperative laboratory data included C-reactive protein level, erythrocyte sedimentation rate, hemoglobin, hematocrit, and platelet concentrations; total lymphocyte count, albumin level, prealbumin level, and coagulation test results (prothrombin time [PT], prothrombin activity [PA], international normalized ratio [INR], activated partial thromboplastin time [APTT], D-dimer level, and fibrinogen concentration). All data were collected from medical records.

For patients who were preoperatively using aspirin or clopidogrel, we performed the procedure at 1 week after discontinuation. Experienced senior surgeons performed all operations. Antibiotics were routinely administered at 30 min before incision and for 24–72 h after surgery to prevent infection. A drainage tube was routinely placed in all patients and removed within 48 h postoperatively. Data on postoperative drainage volume were obtained from medical records, which were originally recorded by nurses. To prevent deep vein thrombosis, all patients were routinely administered antiembolism stockings and anticoagulation drugs.

Based on our institutional policy, the allogeneic blood transfusion triggers were as follows: hemoglobin concentration < 7.0 g/dL and hematocrit < 25%, or hemoglobin concentration < 8.0 g/dL for patients aged > 60 years with cardiovascular disease, or symptoms of acute anemia (including an altered mental status, low blood pressure, pallor, and shortness of breath not attributable to other causes).

### Statistical analysis

Demographic data were compared between patients who received an allogeneic blood transfusion and those who did not. The normality of data distribution was evaluated using the Shapiro–Wilk test. Normally distributed continuous data are expressed as mean ± standard deviation (SD) and were compared using Student’s t test. Non-normally distributed continuous data are presented as median (interquartile range [IQR]) and were compared using the Mann–Whitney U test. Categorical data are expressed as frequencies and percentages and were compared using the Pearson chi-square test or Fisher’s exact test. Multivariate logistic regression analysis was conducted to identify factors associated with perioperative blood transfusion. A stepwise logistic model was used to determine the best-fit multivariate model.

All statistical analyses were performed using SPSS software version 23.0 (IBM Corp., Armonk, NY, USA). Statistical significance was set at *P* < 0.05 (two-sided).

## Results

A total of 331 patients (290 [87.6%] men, 41 [12.4%] women) were included in this study. The mean age at operation was 41.5 ± 13.4 years, and the mean BMI was 23.4 ± 4.4 kg/m^2^. Overall, 113 patients (34.1%) received perioperative allogeneic blood transfusion. Among them, 10 (8.8%) received intraoperative transfusion, 85 (75.2%) received postoperative transfusion, and 18 (15.9%) received intraoperative and postoperative transfusions. The median transfusion volume was 4 units (IQR 2–4).

Demographic data, clinical parameters, and operation-related data are presented in Table 1, and the preoperative laboratory data are shown in Table 2. In the univariate analysis, female sex (*P* = 0.035), lower BMI (*P* = 0.006), ankylosing hip (*P* < 0.001), prolonged operative duration (*P* < 0.001), greater estimated intraoperative blood loss (*P* < 0.001), greater intraoperative autogenic transfusion volume (*P* < 0.001), and greater postoperative drainage volume (*P* < 0.001) were associated with an increased risk of allogeneic blood transfusion. Perioperative TXA use (*P* < 0.001) was associated with a lower risk of allogeneic blood transfusion.

**Table 1:** Demographic and intraoperative data of the study population

Variable	Transfusion (*n* = 113)	Non-transfusion (*n* = 218)	*P* value
Female, n (%)	20 (17.7%)	21 (9.6%)	0.035
Age at THA, years (median, IQR)	40 (31–53)	40 (31–51)	0.761
BMI, kg/m^2^ (median, IQR)	22.0 (18.7–25.4)	23.9 (21.1–26.6)	0.006
Disease duration, years (median, IQR)	16 (11–25)	15 (10–25)	0.110
Preoperative BASDAI (median, IQR)	3.7 (3.0–5.3)	3.9 (3.1–5.1)	0.661
Preoperative BASFI (median, IQR)	5.4 (4.5–6.2)	5.4 (4.3–6.4)	0.420
Preoperative Harris score (median, IQR)	39 (30–46)	32 (39–45)	0.926
ASA score ≤ 2, n (%)	104 (92.0%)	196 (89.9%)	0.529
Ankylosing hip, n (%)	52 (46.0%)	60 (27.5%)	< 0.001
Perioperative TXA use, n (%)	41 (36.3%)	192 (88.1%)	< 0.001
General anesthesia, n (%)	90 (79.6%)	159 (72.9%)	0.180
Operative duration, minutes (median, IQR)	90 (80–120)	78 (60–90)	< 0.001
Estimated intraoperative blood loss, mL (median, IQR)	600 (500–1000)	400 (200–500)	< 0.001
Intraoperative autogenic transfusion volume, mL (median, IQR)	240 (200–400)	100 (0–200)	< 0.001
Postoperative drainage volume, mL (median, IQR)	230 (50–400)	30 (0–200)	< 0.001

*ASA* American Society of Anesthesiologists, *BASDAI* Bath Ankylosing Spondylitis Disease Activity Index, *BASFI* Bath Ankylosing Spondylitis Functional Index, *BMI* body mass index, *IQR* interquartile range, *THA* total hip arthroplasty, *TXA* tranexamic acid

**Table 2:** Preoperative laboratory data of the study population

Variable	Transfusion (*n* = 113)	Non-transfusion (*n* = 218)	*P* value
CRP, mg/L (median, IQR)	12.8 (5.3–24.9)	11.9 (5.7–22.1)	0.919
ESR, mm/h (median, IQR)	19 (9–36)	19 (11–34)	0.940
Hemoglobin, g/dL (median, IQR)	13.7 (12.6–14.7)	14.3 (13.3–15.2)	0.001
Hematocrit, % (mean ± SD)	41.0 ± 4.6	42.7 ± 4.1	0.001
Albumin, g/L (median, IQR)	44.1 (41.0–45.6)	45.0 (42.1–47.1)	0.009
Prealbumin, mg/L (median, IQR)	188.5 (154.3–232.5)	216.0 (177.3–247.5)	< 0.001
PT, s (median, IQR)	11.7 (11.2–12.4)	12.2 (11.6–12.8)	< 0.001
PA, % (median, IQR)	89.3 (83.8–96.8)	98.2 (88.7–105.0)	< 0.001
INR (median, IQR)	1.04 (0.99–1.10)	1.01 (0.96–1.05)	0.001
APTT, s (median, IQR)	29.4 (26.7–32.7)	29.2 (27.0–32.8)	0.803
D-dimer, mg/L FEU (median, IQR)	0.36 (0.18–0.97)	0.37 (0.23–0.75)	0.900
Fibrinogen, mg/dL (median, IQR)	393.9 (331.6–447.7)	369.4 (321.1–426.5)	0.252

*APTT* activated partial thromboplastin time, *CRP* C-reactive protein, *ESR* erythrocyte sedimentation rate, *INR* international normalized ratio, *IQR* interquartile range, *PA* prothrombin activity, *PT* prothrombin time, *SD* standard deviation

There were significant differences in preoperative hemoglobin (*P* = 0.001), hematocrit (*P* = 0.001), albumin (*P* = 0.009), prealbumin (*P* < 0.001), PT (*P* < 0.001), PA (*P* < 0.001), and INR (*P* = 0.001) between the two patient groups.

In the multivariate logistic regression analysis, prolonged operative duration (odds ratio [OR] per 10 min = 1.139, 95% confidence interval [CI] = 1.002–1.294, *P* = 0.047), increased estimated intraoperative blood loss (OR per 100 mL = 1.348, 95% CI = 1.188–1.530, *P* < 0.001), and increased postoperative drainage volume (OR per 100 mL = 1.235, 95% CI = 1.028–1.483, *P* = 0.024) were risk factors for transfusion. In addition, higher BMI (OR = 0.914, 95% CI = 0.852–0.981, *P* = 0.012), perioperative TXA use (OR = 0.166, 95% CI = 0.085–0.326, *P* < 0.001), and higher preoperative hemoglobin level (OR per 1 g/dL = 0.744, 95% CI = 0.607–0.912, *P* = 0.004) were protective factors against the need for transfusion (Table 3). The multivariate model was accurate for 81.8% of all patients, and a concordance ratio greater than 75% was considered to indicate that the prediction model was highly accurate.

**Table 3:** Multivariate logistic regression analysis for transfusion in THA

	OR	95% CI	Change	*P* value
Operative duration	1.139	1.002–1.294	Δ10 min	0.047
Estimated intraoperative blood loss	1.348	1.188–1.530	Δ100 mL	< 0.001
Postoperative drainage volume	1.235	1.028–1.483	Δ100 mL	0.024
Body mass index	0.914	0.852–0.981	Δ1 kg/m^2^	0.012
Perioperative TXA use	0.166	0.085–0.326		< 0.001
Preoperative hemoglobin level	0.744	0.607–0.912	Δ1 g/dL	0.004

*CI* confidence interval, *OR* odds ratio, *THA* total hip arthroplasty, *TXA* tranexamic acid

## Discussion

In this study, 34.1% of patients with AS received an allogeneic blood transfusion during primary unilateral THA. We found that prolonged operative duration, increased estimated intraoperative blood loss, and increased postoperative drainage volume were risk factors for transfusion, whereas a higher BMI, perioperative TXA use, and higher preoperative hemoglobin level were protective factors. Our study is valuable because it identified risk factors for transfusion using logistic regression analysis, with a relatively large sample size.

A large database analysis indicated that the overall allogeneic blood transfusion rate was 22.1% among Chinese patients undergoing primary THA [8]. In other countries, the transfusion rate has been reported to range from 2.5 to 49.9% [15–18]. In patients with AS undergoing THA in China, the transfusion rate has been reported to range from 73.3 to 87.5% [4–6]. However, the transfusion rate in our study was lower than that previously reported. This difference may be attributed to the various transfusion triggers among different institutions. Furthermore, we also found that the transfusion rate decreased with time, dropping from 60.9% between 2011 and 2015 to 14.7% between 2016 and 2021. This may be attributed to improvements in surgical techniques and perioperative management [18].

Previous studies have reported female sex as a risk factor for transfusion [7, 9, 10, 15–25]. Our univariate analysis yielded similar results; nonetheless, these failed to remain significant in the multivariate logistic model. Female sex may be associated with other factors, such as lower BMI and lower preoperative hemoglobin levels. Considering the confounding potential arising from the low proportion of female patients with AS, studies involving more female patients need to be conducted.

Our results showed that the risk of receiving a transfusion decreased with increasing BMI, which is consistent with previous findings [17–20, 22–24]. Previous studies have found that BMI was lower in patients with AS than in patients with osteoarthritis [5, 26]. Zhao et al. found that the incidence of underweight (BMI < 18.5 kg/m^2^) was 39% (91/236) in patients with AS undergoing THA and that the transfusion rate was significantly higher in the underweight group (82.4% vs. 67.6%) [4]. Patients with a higher BMI have a higher estimated blood volume, which results in a lower final percentage of blood volume loss during THA [27], which may reduce the need for transfusion. In our study, the prevalence of underweight was 15.1%, and the transfusion rate was not significantly higher in the underweight group.

The disease activity of AS is reported to influence blood loss during THA [28, 29]. Patients with active disease lost 600 mL more blood perioperatively than patients with stable disease [28]. Higher levels of inflammation, which suggest greater disease activity, are associated with anemia and local imbalances in coagulation and fibrinolysis activation, which may lead to greater blood loss [28, 30]. However, there was no significant difference in the transfusion rate between the two groups [28]. Similarly, we found that disease activity was not associated with the need for transfusion; the disease activity of most patients was well controlled in our study, which may explain the results.

Compared with patients with AS and stiff hips, patients with AS and ankylosing hips had a higher perioperative transfusion rate [5]. There were no differences in blood loss and transfusion rate between patients with AS with stiff hips and those with osteoarthritis [5]. Li et al. stated that the difficulty and duration of the operation were the main risk factors for increased blood loss [5]. However, Li et al. only included 40 cases in each group, and the intraoperative transfusion rate of patients with osteoarthritis was relatively high (76.3%). In our study, we found that a bony ankylosing hip was associated with transfusion during THA. Given that this factor was not significant in the multivariate model, additional studies are needed to investigate this potential association.

Consistent with previous studies, we found that prolonged operative duration was a risk factor for transfusion in THA [6, 7, 18, 20, 23]. This may be attributed to the complexity of the surgery. For each additional 10 min of operative time, the risk of receiving blood transfusion increased by 13.9%. Surgeons should strive to shorten the operation time to reduce the risk of transfusion.

We found that patients receiving transfusion had increased intraoperative blood loss, intraoperative autogenic transfusion volume, and postoperative drainage volume. Increased intraoperative blood loss and increased postoperative drainage volume were also identified as risk factors in the multivariate logistic model. Excessive blood loss leads to a higher risk of perioperative transfusion, as reported by previous studies [7, 8, 17, 20, 22]. Therefore, it is important to reduce intraoperative blood loss to decrease the need for perioperative transfusion.

Perioperative TXA use is a clinically and cost-effective method for reducing blood loss during THA [6, 31, 32]. Our results showed that the use of TXA was associated with an 83% decreased risk of transfusion, which is consistent with previous findings [6, 8, 23, 31, 33]. TXA is a derivative of lysine, which can inhibit fibrin from binding to plasminogen, leading to an antifibrinolytic effect [34]. A network meta-analysis similarly showed that TXA led to a significant reduction in perioperative blood loss and a lower risk of transfusion [31].

Consistent with previous reports, we found that a lower preoperative hemoglobin level was associated with a high risk of transfusion [7–9, 19, 20, 22–25, 33, 35]. Preoperative anemia is a modifiable risk factor for blood transfusion and other postoperative complications [32, 33]. Klement et al. reported that preoperative hemoglobin levels of 12.5 g/dL for women and 13.5 g/dL for men comprised the optimal cutoff values to predict the need for transfusion [33]. Patients with values lower than these may benefit from preoperative optimization of hemoglobin levels to decrease their risk of transfusion. Thus, we recommend preoperative detection and treatment of anemia before THA for patients with AS. In our study, there were some differences in albumin and prealbumin levels and coagulation test results between the two groups. However, using the forward likelihood ratio method, these factors were not included in the multivariate model. Lower albumin and prealbumin levels may indicate poor preoperative nutritional status, and previous studies have reported that hypoalbuminemia is a risk factor for transfusion [36, 37]. Moreover, Shi et al. found that patients with AS had higher levels of APTT, PT, and INR, with a lower level of fibrinogen [26], whereas another study showed that there was no difference in preoperative coagulation test results between patients with osteoarthritis and those with AS [5]. Two other studies reported that patients with AS had higher D-dimer levels [38, 39]. AS is a disease that affects multisystem functions, including coagulation [26]. Given that these laboratory tests may be associated with other factors, further studies are needed to investigate these.

This study had several limitations. This was a retrospective observational study with no control group. The study period spanned a long period, and the transfusion rate decreased with advancements in surgical techniques and perioperative management. However, our study included a relatively large sample size of patients with AS. Second, there was no information on bone mineral density or selective serotonin reuptake inhibitor utilization in the medical records, which may have also affected the results [15, 40, 41]. Intraoperative blood loss was estimated by the surgeon, which may have caused data inaccuracy. In addition, the operations were performed by multiple surgeons. However, they were all experienced senior surgeons who had received standard orthopedic training. Prospective studies with larger sample sizes are needed to determine the association between these factors and the transfusion rate.

## Conclusions

Allogeneic blood transfusion was associated with worse surgical and medical outcomes, as well as a longer hospital stay after THA [9, 10, 15]. In addition, patients receiving allogeneic blood transfusion are at a higher risk for the development of surgical-site infections [12, 20]. We found that prolonged operative duration, increased estimated intraoperative blood loss, and increased postoperative drainage volume were risk factors for transfusion, whereas higher BMI, perioperative TXA use, and higher preoperative hemoglobin level were protective factors. The transfusion rate decreased over time, which may be attributed to advancements in surgical techniques, perioperative management, and control of disease activity by internal medicine. These results may aid in the development of a better preoperative plan and perioperative management strategy, ultimately reducing the need for transfusion.

## Data Availability

The datasets used and/or analyzed during the current study are available from the corresponding author on reasonable request.

## Abbreviations

APTT: Activated partial thromboplastin time
AS: Ankylosing spondylitis
BMI: Body mass index
CI: Confidence interval
INR: International normalized ratio
IQR: Interquartile range
OR: Odds ratio
PA: Prothrombin activity
PT: Prothrombin time
SD: Standard deviation
THA: Total hip arthroplasty
TXA: Tranexamic acid
